# Solution-Processed Mesoscopic Bi_2_S_3_:Polymer Photoactive Layers

**DOI:** 10.1002/cphc.201301103

**Published:** 2014-03-05

**Authors:** Andrew J MacLachlan, Flannan T F O'Mahony, Anna L Sudlow, Michael S Hill, Kieran C Molloy, Jenny Nelson, Saif A Haque

**Affiliations:** [a] Department of Chemistry and Centre for Plastic Electronics, Imperial College London Imperial College Road, London, SW7 2AZ (UK) E-mail: s.a.haque@imperial.ac.uk; [b] Department of Chemistry, University of Bath Claverton Down, Bath, BA2 7AY (UK); [c] Department of Physics and Centre for Plastic Electronics, Imperial College London Imperial College Road, London, SW7 2AZ (UK)

**Keywords:** bismuth sulfide, hybrid photovoltaics, nontoxic materials, solution processing, transient absorption spectroscopy

## Abstract

The fabrication of solution-processed nontoxic mesoporous Bi_2_S_3_ structures is demonstrated and the suitability of these structures for use in hybrid solar cells investigated. Mesoporous Bi_2_S_3_ electrodes are prepared via thermal decomposition of a thin film composed of a bismuth xanthate single source precursor. The resultant Bi_2_S_3_ films are made up of regular needles with approximate dimensions of 50×500 nm, as confirmed by scanning electron microscopy (SEM). The crystallinity of the Bi_2_S_3_ is found to be dependent on the annealing temperature, as determined by X-ray diffraction. The porous Bi_2_S_3_ films are infiltrated with the hole conductor P3HT to generate novel hybrid films, and laser-based transient absorption spectroscopy is used to interrogate the charge-separation reaction at the resulting Bi_2_S_3_/P3HT heterojunction. Specifically, optical excitation of the hybrid films results in efficient and long-lived charge separation (microsecond to millisecond timescale), thereby rendering such films suitable for the development of novel low-cost solar-energy conversion devices.

Hybrid photovoltaics have seen a marked increase in power conversion efficiency in recent years.[1] Their attractiveness comes from the ability to combine the superior properties of inorganic materials, such as increased charge carrier mobilites, high dielectric permittivity, enhanced chemical stability and cheapness to produce, along with the processing advantages that have made organic photovoltaics seem so desirable. Work on hybrid photovoltaics to date has utilised a wide range of inorganic materials within the active layer including CdS,[2] CdSe,[3] Sb_2_S_3_,[4] PbS,[5] CuInS_2_[6] and ZnO.[7] Challenges to the design of efficient hybrid solar cells are well known and include: 1) the development of alternative inorganic electron acceptors to the highly toxic materials that have thus far been successful in hybrid solar cells (e.g. Pb or Cd based) and 2) the development of solution-based processing routes that enable the design of donor–acceptor hybrid inorganic–organic photoactive layers exhibiting high yields of charge photogeneration and efficient carrier collection at the device electrodes. To address challenge (2) we recently reported a new approach to the design of hybrid films based upon the in situ growth of metal sulfide nanocrystals in semiconducting polymer films using metal xanthate (or metal *o*-alkyl dithiocarbonate) precursors.[8] This approach has been shown to be an effective method for the production of CdS,[9] Sb_2_S_3_[4] and CuInS_2_[6] polymer bulk heterojunction solar cells. An alternative approach to the preparation of hybrid films is to use a pre-formed mesoporous inorganic semiconductor film as a functional scaffold, which is subsequently infiltrated by an appropriate organic semiconductor to generate an inorganic-organic nanocomposite assembly. This method offers two main advantages; better definition of connectivity by ensuring a continuous phase before the addition of a polymer and also the ability to tune the chemical/physical properties of the interface between the two phases. To this end, we have recently demonstrated that metal xanthate precursors can be used to fabricate three-dimensional mesoporous metal sulfide films.[10] Specifically, we demonstrated the fabrication of mesoporous Sb_2_S_3_ films where we were able to control the mesoporosity of the films through tuning the annealing temperature and were able to show the dependence of charge generation on the interfacial area along with working photovoltaic devices.[10] Here we extend this approach to the fabrication of mesoporous Bi_2_S_3_ layers. Bi_2_S_3_ is particularly attractive for use as an electron acceptor and light absorber in hybrid solar cells due to its low toxicity, low bandgap (1.3–1.7 eV),[11] which facilitates a broad coverage of the solar spectrum, a reasonably strong absorption coefficient (∼10^5^ cm^−1^)[12] and high electron mobility (28 cm^2^ V^−1^ s^−1^).[13] In this work, our approach is based on the thermal decomposition of a bismuth xanthate precursor film. Control of the crystallinity of the resultant Bi_2_S_3_ films is demonstrated by varying the temperature at which the precursor film is annealed. Transient absorption spectroscopy (TAS) is employed to study the photoinduced electron and hole transfer at the heterojunction formed by infiltrating the Bi_2_S_3_ structure with P3HT polymer.

The fabrication steps for the preparation of the mesoporous Bi_2_S_3_ films are depicted in Figure 1. The precursor, bismuth triethyldithiocarbonate (BiEX_3_), is first spin-coated onto a ZnO-coated glass substrate from a chlorobenzene solution and then annealed in a nitrogen environment at moderate temperatures (>150 °C). Films are then treated with pyridine, a ligand typically used with nanoparticles to passivate surface traps,[14] followed by the spin coating of the semiconducting polymer P3HT followed by thermal annealing (160 °C). The amount of P3HT used was such that as small a standing layer of P3HT was deposited on top of the mesoporous structures as possible whilst still having full coverage; to best mimic a potential device structure. The absorptance of a typical film after annealing can be seen in Figure 2 a. The Bi_2_S_3_ films can be seen to be highly absorbing across the visible range with an onset of ∼990 nm, as calculated using a Tauc Plot (Supporting Information, SI, Figure S1). Raman spectroscopy confirms the presence of crystalline Bi_2_S_3_ (Figure 2 b), with vibrational modes at 188, 236 and 255 cm^−1^ in agreement with values previously reported.[15] Figure 2 c shows a top down scanning electron microscopy (SEM) image of a film after the decomposition of the precursor, revealing a network of Bi_2_S_3_ nanoneedles with dimensions of approximately 50×500 nm. Interestingly, this morphology appears suited for a hybrid heterojunction as a high surface area is generated while still retaining good connectivity.

**Figure 1:**
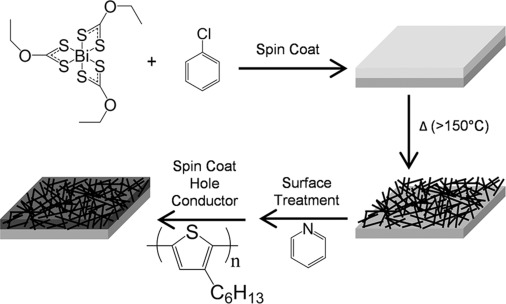
Schematic diagram of heterojunction formation, showing the formation of the mesoporous absorbing layer after the spin coating and annealing of a soluble bismuth xanthate precursor (BiEX_3_), followed by a surface treatment and filling of the pores with a hole conducting polymer.

**Figure 2:**
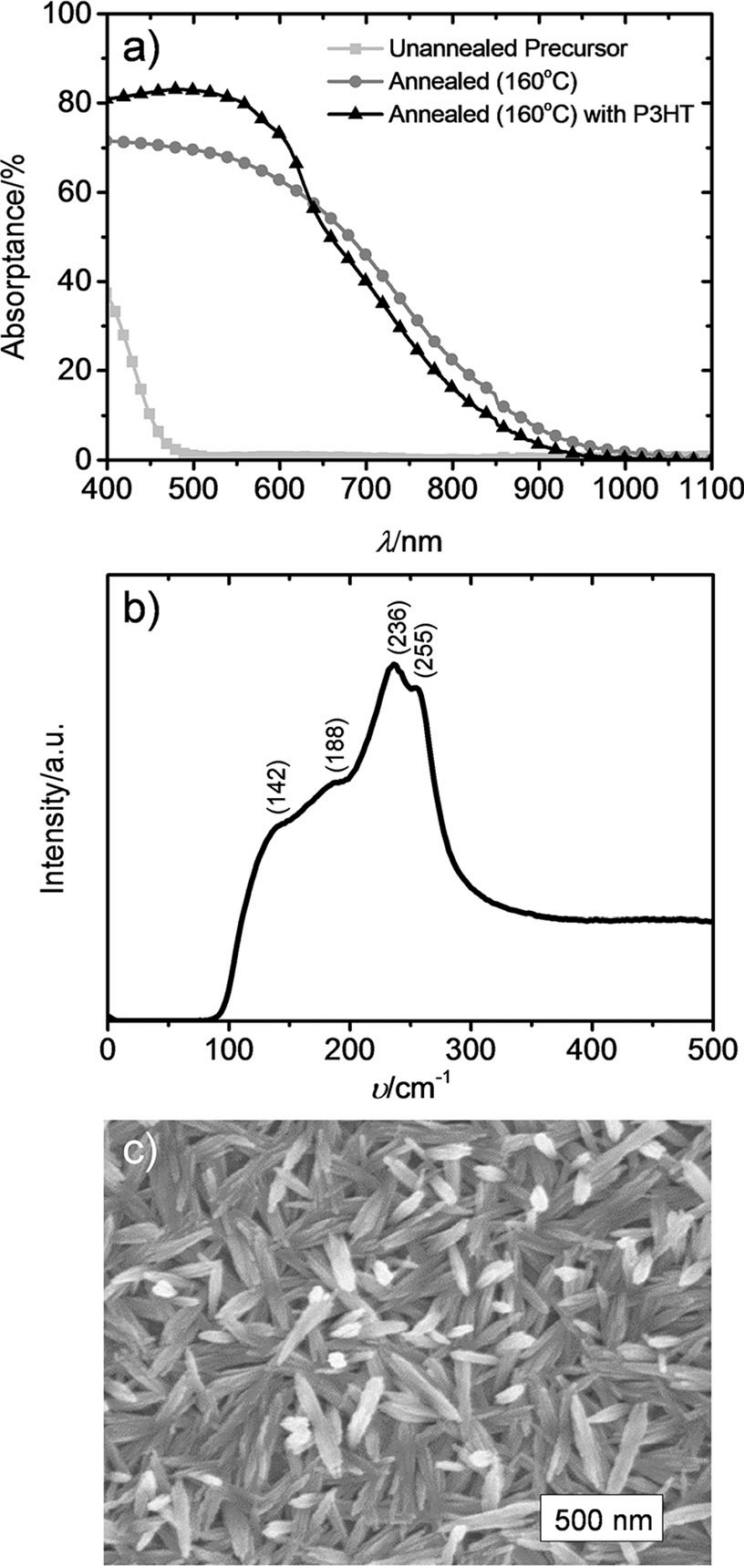
a) Absorptance of a thin film of precursor (grey squares), Bi_2_S_3_ after annealing at 160 °C (dark grey circles) and with a layer of P3HT in the pores (black triangles). b) Raman spectrum and c) top down scanning electron microscopy (SEM) image of a mesoporous Bi_2_S_3_ film.

X-ray diffraction (XRD) was performed on the samples to further confirm the presence of Bi_2_S_3_ and to analyse the crystallinity of the films produced. Figure 3 a shows the XRD pattern for a typical film annealed at 300 °C, along with the reference pattern for bismuthinite directly below.[16] Some of the XRD peaks presented in Figure 3 a appear broad due to a similar scattering angle of certain planes and as a result are a combination of the signal from two crystallographic planes. However, the 220 plane is easily resolved and the Scherrer equation can be used to determine the average crystallite size in this plane. The results of this analysis are shown in Figure 3 b for a range of decomposition temperatures. The Bi_2_S_3_ formed is extremely crystalline, even at low temperatures, with an average particle size of 21.1 nm after annealing at a relatively low temperature of 160 °C. We note that when compared to the SEM image in Figure 2 c it is clear that these needles are comprised of several smaller crystallites. The degree of crystallinity is somewhat dependent on the decomposition temperature used, with a higher annealing temperature resulting in a larger average crystallite size, but this change is relatively small. It is pertinent to note that the average crystallite size observed herein is large and therefore not in the regime where we would expect to see confinement effects. This is supported by the lack of shift in the absorption onset with changing crystallite size (SI, Figure S2). Moreover, it has been previously reported that confinement effects are only observed for particles smaller than 5 nm.[17] The effect of annealing temperature is also visible on the top down SEMs (SI, Figure S3) where the film morphology changes from a network of sharp needles to smoother conjoined rods as the crystallite size is increased.

**Figure 3:**
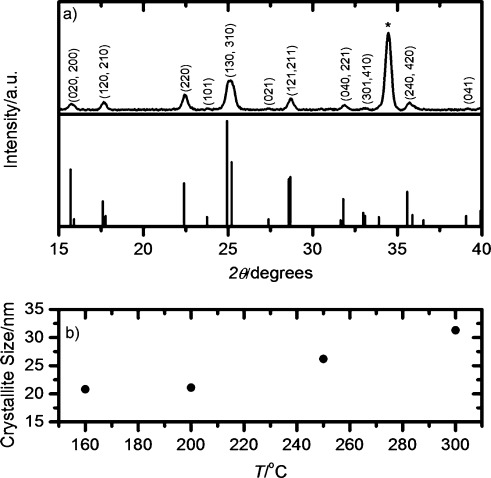
a) X-ray diffraction pattern of a Bi_2_S_3_ film after annealing at 300 °C. * denotes a peak due to ZnO. b) Average crystallite sizes of Bi_2_S_3_ films annealed at different temperatures.

We now consider the photoinduced charge-transfer reactions occurring at the heterojunction between Bi_2_S_3_ and the conjugated polymer P3HT, which was prepared by infiltrating the porous needle network with the polymer. To test the suitability of these heterojunctions for use in photovoltaic devices, transient absorption spectroscopy (TAS) was performed to probe long-lived interfacial charge separation. A transient absorption spectrum was first obtained by exciting the sample using a wavelength of 700 nm, which is only absorbed by the Bi_2_S_3_ phase within the hybrid film, and probing the photoinduced change in absorbance over a range of wavelengths (Figure 4 a). A peak is observed at ∼1000 nm, where P3HT hole polarons typically absorb.[18] At wavelengths below 1000 nm, a bleach is observed, which is likely to be due to depopulation of the ground-state of Bi_2_S_3_. This bleach probably masks the expected second peak of P3HT previously observed at ∼800 nm and may also overlap slightly with the P3HT polaron peak at 980 nm, reducing the magnitude of the signal observed. The relative magnitudes of signals in this case are therefore more important than the actual size, as the apparent yield of P3HT polarons will be consistently reduced by the Bi_2_S_3_ ground state bleach. In Figure 4 b, where only the Bi_2_S_3_ is being excited, there are two clear observations. The first is that although the amplitudes of the lowest 3 temperatures are approximately similar, the 300 °C sample is slightly higher in amplitude, which indicates a higher number of charges in the film 1 μs after photoexcitation. The second observation is that as the annealing temperature is increased the rate of decay and therefore the rate of recombination is also increased. This change in lifetime is approximately an order of magnitude different for the 160 °C and 300 °C samples. The reasons for these observations are not fully understood but we can speculate are likely to be associated with the changing crystallinity of the samples. We have previously shown that the degree of crystallinity in one component of the heterojunction directly affects the yield of long-lived charges generated at the interface, with an increase in crystallinity resulting in a higher yield of charges and a switching on of charge generation in systems with no driving force for separation.[19] In this instance we can speculate that the increase in the average crystallite size and quality is causing the increase in yield of charges due to the effect of an increased delocalisation of charges generated in the Bi_2_S_3_. Bi_2_S_3_ has previously been shown to have a high electron mobility[13] and an increase in crystallinity is likely to result in a higher mobility. An increased mobility of electrons in Bi_2_S_3_ could result in an increase in the rate of non-geminate recombination after charge separation, due to the electrons having a greater ability to diffuse through the film. Such behaviour is often observed in all-organic photovoltaic systems.[20] It is also pertinent to note that without the treatment of pyridine almost no signal was observed (SI, Figure S4). In this system it is clear that control of the inorganic–organic interface is critical to the performance of the heterojunction. In Figure 4 c, where both the P3HT and Bi_2_S_3_ are being excited, the signal intensities are seen to be lower than in the previous case but with similar lifetime. This is an indication of the lack of charge generation from P3HT in comparison to charge generation from Bi_2_S_3_. The samples were excited in both cases through the P3HT layer, so the lack of generation from the polymer could be due to the layer of P3HT being too thick to allow for exciton diffusion to the interface. Alternatively, the exciton splitting could be inefficient in this system. We are unable to determine which of these explanations is correct, however this observation reiterates the need to find a wider bandgap polymer that does not filter light and hinder the maximum currents possible from these types of devices, as previously reported.[10]

**Figure 4:**
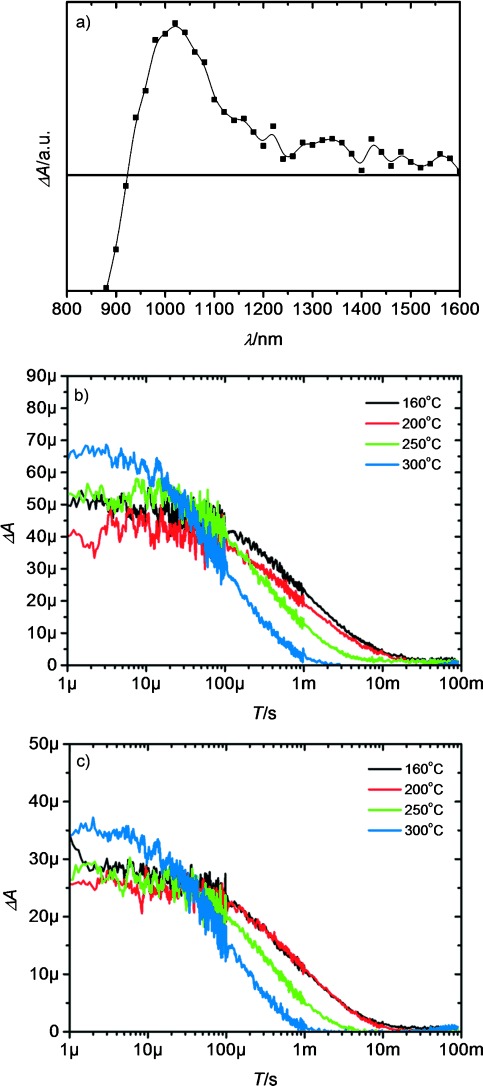
a) Transient absorption spectrum of a Bi_2_S_3_:P3HT heterojunction. Kinetic decays of the P3HT hole polaron after 700 nm (b) and 510 nm (c) excitation. Laser fluencies used were 6.97 (b) and 9.70 μJ cm^−2^ (c) keeping the amount of excitation photons approximately the same. All samples were corrected for the number of photons absorbed.

In conclusion, we have demonstrated a simple processing route to nontoxic mesoporous Bi_2_S_3_ films, in which a high level of crystallinity is achieved at relatively low temperatures. The resulting films are extremely absorbing with a band gap of approximately 990 nm. When the pores have been filled with the hole conducting polymer P3HT, the films have been shown to photogenerate charges across the inorganic/organic interface as determined by transient absorption spectroscopy. With further studies currently underway we expect that this route could prove useful for producing high efficiency nontoxic solution-processed photovoltaic devices.

## Experimental Section

### Synthesis of Bismuth(III)tri(O-ethyldithiocarbonate) (BiEtX_3_)

The syntheses of this compound and other bismuth *o*-alkylthiocarbonates have been well established previously. Typically, Bi(NO_3_)_3_⋅5 H2O (5.0 g, 10.3 mmol) was suspended in aqueous solution (100 mL) and conc. HCl was added dropwise until a clear solution was obtained. Potassium ethylxanthogenate (5.0 g, 31.2 mmol) was dissolved in water (25 mL) and added to the bismuth nitrate solution and stirred for 30 min at room temperature, RT. The yellow solid formed was collected by vacuum filtration and recrystallised from a chloroform solution. ^1^H NMR (400 MHz, CDCl_3_): *δ*=4.70 (q, 2 H, CH_2_), 1.50 ppm (t, 3 H, CH_3_). Elemental analysis (%) found: C, 19.00; H, 2.74. Calc: C, 18.88; H, 2.64.

### Characterisation

Steady-state absorption measurements were carried out with a Shimadzu 2600 spectrophotometer with an ISR-2600Plus Intergrating Sphere Attachment.

Raman spectroscopy was performed using a Renishaw inVia Raman microscope in a backscattering configuration with a 514 nm Ar laser excitation.

Transient absorption spectroscopy (TAS) measurements were performed by exciting the sample film under a dynamic nitrogen atmosphere using a dye laser (Photon Technology International Inc. GL-301) pumped by a nitrogen laser (Photon Technology International Inc. GL-3300). A 100 W quartz halogen lamp (Bentham, IL 1) with a stabilized power supply (Bentham, 605) was used as a probe light source, with a probe wavelength of 980 nm used. The probe light passing through the sample film was detected with a silicon photodiode (Hamamatsu Photonics, S1722-01). The signal from the photodiode was amplified before being passed through electronic band-pass filters (Costronics Electronics). The amplified signal was collected with a digital oscilloscope (Tektronics, DPO3012), which was synchronized with a trigger signal from the pump laser pulse from a photodiode (Thorlabs Inc., DET210).

Scanning electron microscopy (SEM) was performed using the In Lens detector of a LEO 1525 field-emission scanning electron microscope operating at 5 kV. Samples were sputter-coated with a ∼17 nm layer of chromium prior to the measurement to improve the conductivity.

X-ray diffraction (XRD) was conducted with a PANalytical X'Pert Pro MRD diffractometer using Ni-filtered Cu K-alpha radiation at 40 kV and 40 mA.

## Supporting Information

As a service to our authors and readers, this journal provides supporting information supplied by the authors. Such materials are peer reviewed and may be re-organized for online delivery, but are not copy-edited or typeset. Technical support issues arising from supporting information (other than missing files) should be addressed to the authors.

miscellaneous_information
