# Supplementary material for: Solution-Processed Mesoscopic Bi2S3:Polymer Photoactive Layers
Source: Chemphyschem. 2014 Mar 5;15(6):1019–23. doi: 10.1002/cphc.201301103 (PMC4501321; doi:10.1002/cphc.201301103)
Supplement: Supplementary file 1 — miscellaneous_information [file cphc0015-1019-sd1.pdf]

CHEMPHYSCHEM

Supporting Information

© Copyright Wiley-VCH Verlag GmbH & Co. KGaA, 69451 Weinheim, 2014

Solution-Processed Mesoscopic Bi_2S_3:Polymer Photoactive Layers

Andrew J. MacLachlan,[a] Flannan T. F. O'Mahony,[a] Anna L. Sudlow,[b] Michael S. Hill,[b] Kieran C. Molloy,[b] Jenny Nelson,[c] and Saif A. Haque*[a]

cphc_201301103_sm_miscellaneous_information.pdf

Supplementary Information

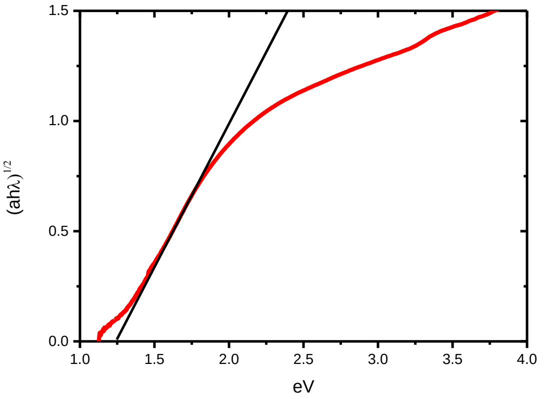

SI Figure 1. Tauc analysis for an indirect bandgap material performed on a typical Bi_2S_3 film giving an estimate for the bandgap as ~1.25 eV or 990 nm.

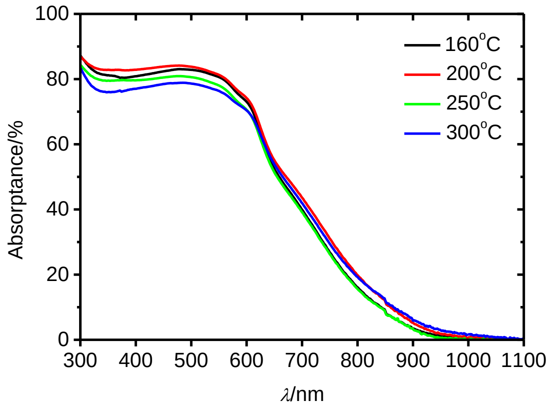

SI Figure 2. Absorptance of films annealed at different temperatures showing no change in bandgap.

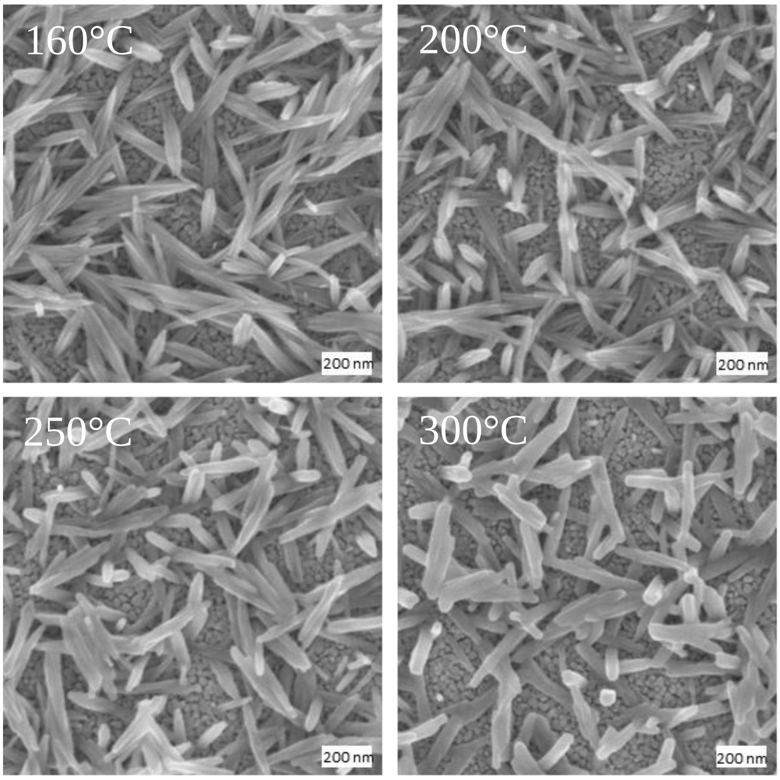

SI Figure 3. Top-down scanning electron microscopy (SEM) images of Bi_2S_3 films after annealing at various temperatures.

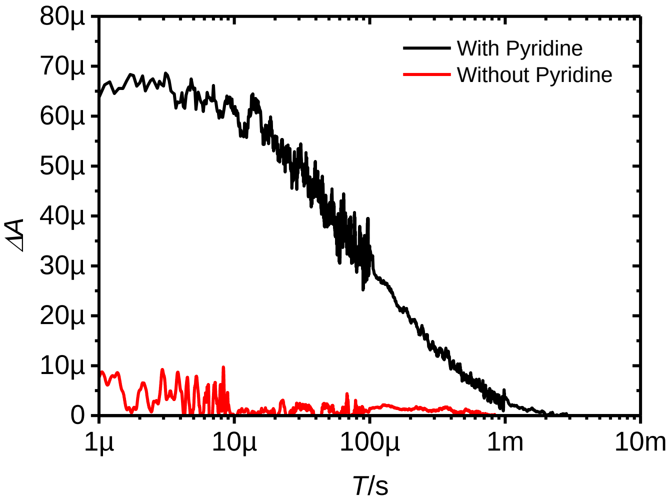

SI Figure 4. Transient absorption kinetic decays of Bi_2S_3 films annealed at 300°C and infiltrated with P3HT with and without a soak in pyridine.

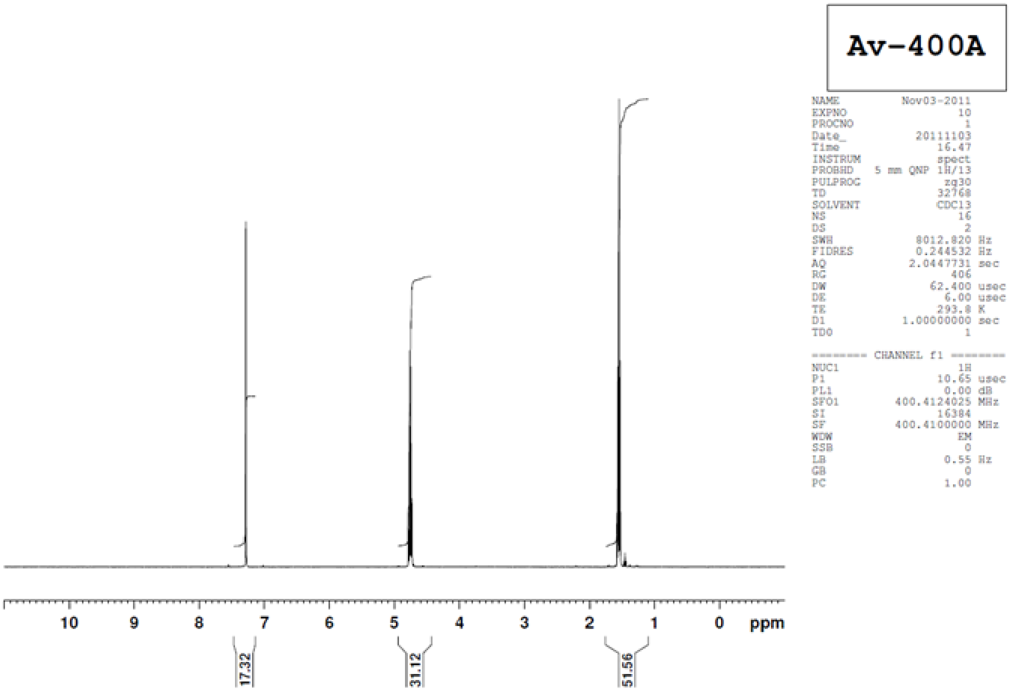

SI Figure 5. ^{1}H NMR of bismuth ethylxanthate in $CDCl_3$
